# Optimization and multiple in vitro activity potentials of carotenoids from marine *Kocuria* sp. RAM1

**DOI:** 10.1038/s41598-022-22897-4

**Published:** 2022-10-28

**Authors:** Rasha A. Metwally, Nermeen A. El-Sersy, Amany El Sikaily, Soraya A. Sabry, Hanan A. Ghozlan

**Affiliations:** 1 Marine Microbiology Lab., National Institute of Oceanography and Fisheries, NIOF, Alexandria, Egypt; 2 Marine Pollution Lab., National Institute of Oceanography and Fisheries, NIOF, Alexandria, Egypt; 3 Botany & Microbiology Department, Faculty of Science, Alexandria University, Alexandria, Egypt

**Keywords:** Biotechnology, Microbiology, Drug development, Natural products

## Abstract

Marine pigmented bacteria are a promising natural source of carotenoids. *Kocuria* sp. RAM1 was isolated from the Red Sea *Bohadschia graeffei* collected from Marsa Alam, Egypt, and used for carotenoids production. The extracted carotenoids were purified by thin-layer chromatography (TLC). The characteristic UV absorbance of the three purified fractions gave us an inkling of what the purified pigments were. The chemical structures were confirmed by nuclear magnetic resonance spectroscopy (NMR) and LC-ESI-QTOF-MS/MS. The three different red pigments were identified as two C_50_-carotenoids, namely bisanhydrobacterioruberin and trisanhydrobacterioruberin, in addition to 3,4,3ʹ,4ʹ-Tetrahydrospirilloxanthin (C_42_-carotenoids). *Kocuria* sp. RAM1 carotenoids were investigated for multiple activities, including antimicrobial, anti-inflammatory, antioxidant, anti-HSV-1, anticancer, antidiabetic and wound healing. These new observations suggest that *Kocuria* sp. RAM1 carotenoids can be used as a distinctive natural pigment with potent properties.

## Introduction

Pigmented bacteria are extremely common in marine habitats. Natural pigments could be used as “green chemistry” to replace synthetic ones^1^. During the period 2020–2027, demand for pigments will increase by 5% per year^2^. Among them, are carotenoids which are thought to be the most important and abundant pigment group^3^. Carotenoids are isoprenoid derivatives found in nature that feature a variety of biological functions^4^. They are characterized by a polyene chain of conjugated double bonds, causing distinctive absorption patterns, imparting colors, and playing potential biological tasks such as cell protection from UV radiation and antioxidant effects^5,6^.

Carotenoids also perform a variety of functions, such as harvesting solar energy, increasing pathogen virulence, modulating the immune system, pro-vitamin A activity and other functional properties^5^, which makes them one of the most important compounds applied industrially in food, cosmetic, and pharmaceutical product formulations^7^.

Upwards of 750 naturally occurring carotenoids have been originated in plants, animals, and microorganisms, which are primarily divided into carotenes (lack of oxygen) and xanthophylls (presence of oxygen)^8^. Microorganisms are now thought to be alternative natural sources of biomolecules with promising industrial biotechnological applications due to their ease of cultivation by applying controlled parameters of nutrients, pH, temperature, and aeration^7^. Thus, the current study sought to identify a new marine pigmented bacterium for carotenoids production as well as to screen for potential biomedical applications.

## Results

### Isolation, screening and identification of the pigmented isolate

**Figure 1:**
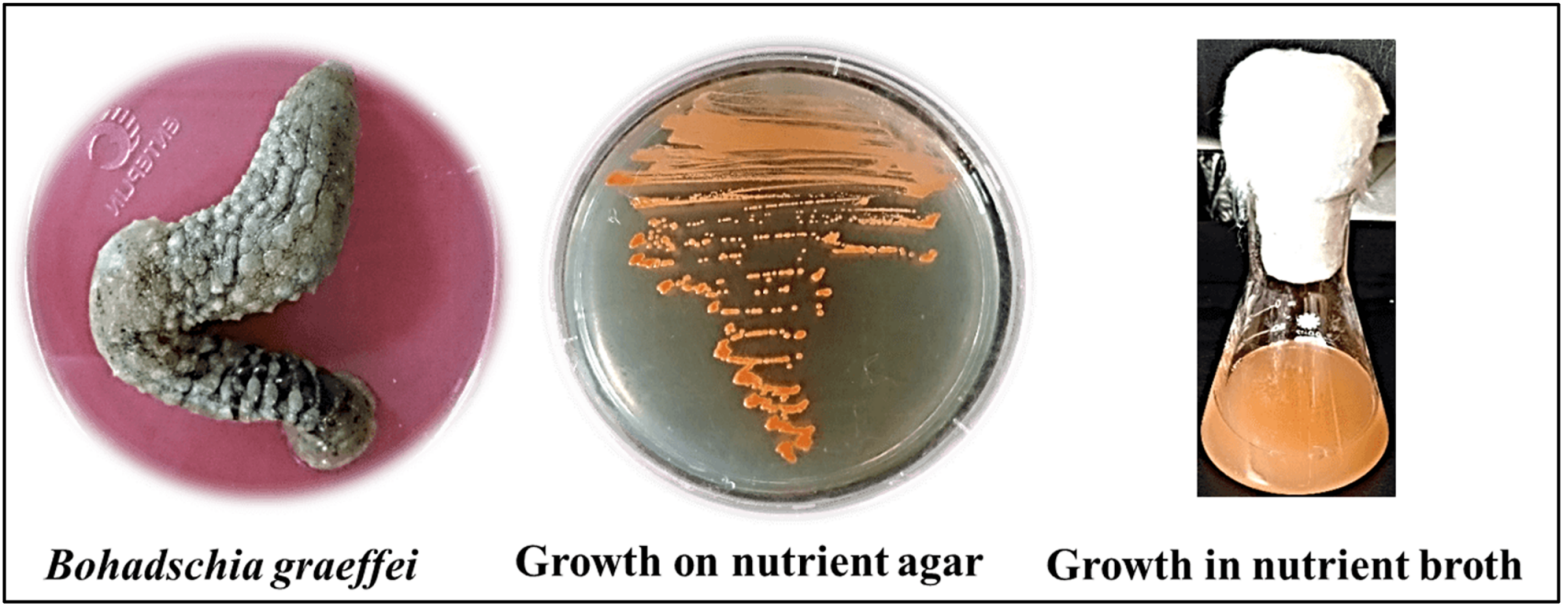
*Kocuria* sp. RAM1 with orange pigmentation isolated from *Bohadschia graeffei,* Marsa Alam, Red Sea, Egypt.

**Figure 2:**
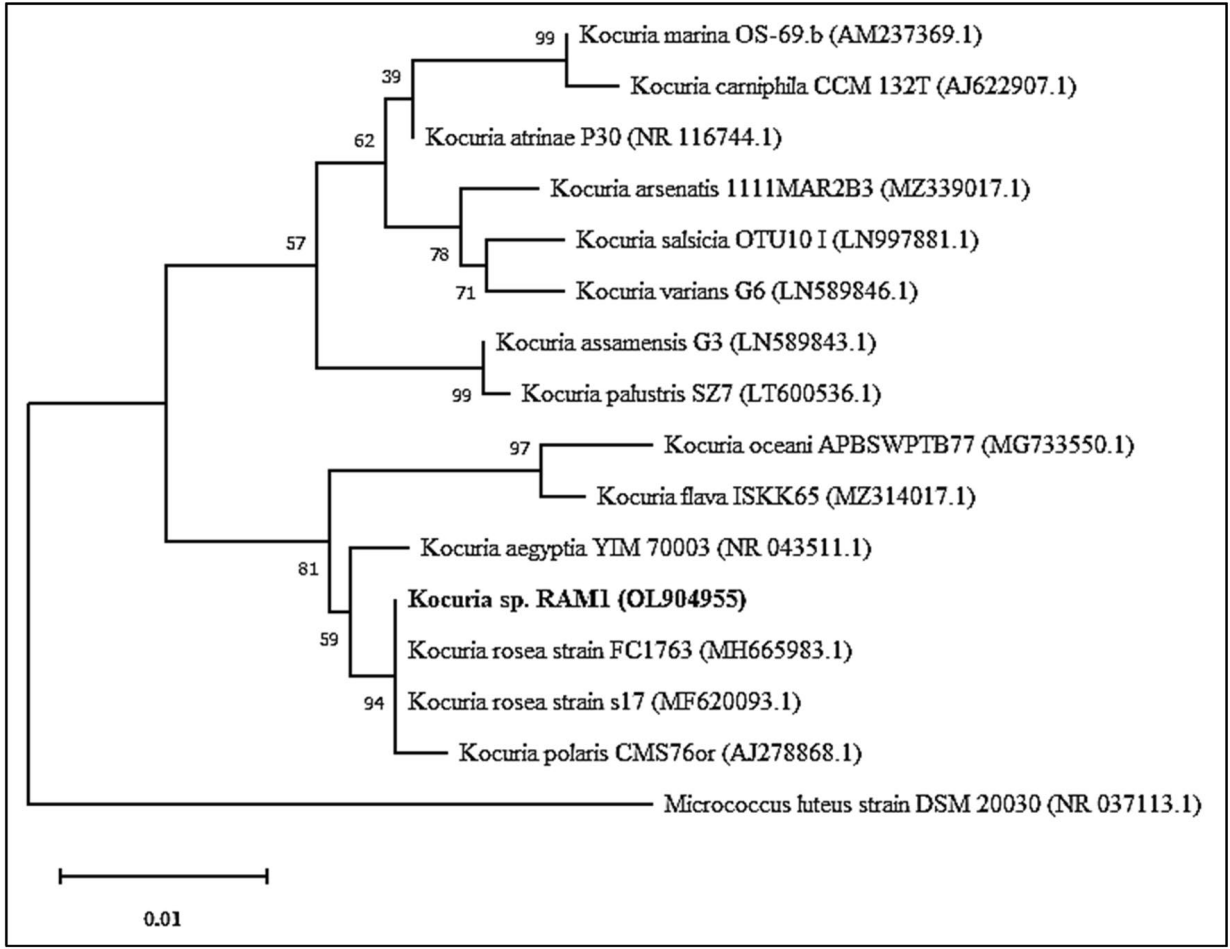
Phylogenetic tree of *Kocuria* sp. RAM1 estimated using maximum likelihood (ML). *Kocuria* sp. RAM1 was indicated by a bold style font. GenBank sequence accession numbers are indicated in parentheses after the strain names. The evolutionary analysis was conducted in the software package MEGA 11.

In the current study, an orange-pigmented bacterial strain was isolated from *Bohadschia graeffei* collected from Marsa Alam, Red Sea, Egypt, purified and plated on nutrient agar medium. Colonies were round, smooth, raised, convex, mucoid and with non-diffusible orange pigmentation (Fig. 1). Cells under the microscope were Gram-positive cocci. The biochemical characterization was recorded in Supplementary Table S1. The isolate was given the name *Kocuria* sp. RAM1 as revealed by molecular identification and submitted to GenBank at NCBI Nucleotide database (Accession Number: OL904955) (Fig. 2).

### Pigment extraction, purification and identification

**Figure 3:**
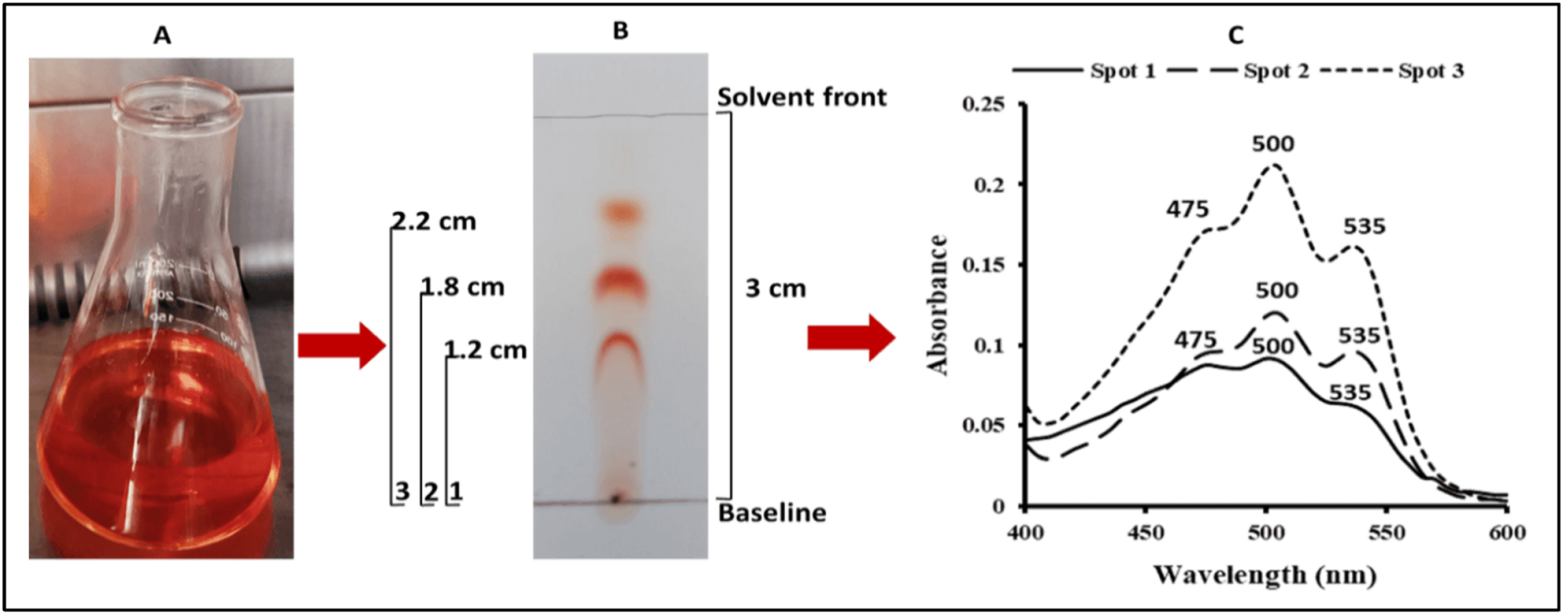
*Kocuria* sp. RAM1 carotenoids. (**A**) Carotenoids dissolved in petroleum ether. (**B**) Thin-Layer Chromatography (TLC) [solvent system = chloroform: methanol (6:1; v/v)]. (**C**) UV–Vis spectra of the 3 purified pigment spots obtained from TLC.

Methanol was found to be the best solvent for the intracellular pigment extraction from *Kocuria* sp. RAM1 cells. Thin-layer chromatography (TLC) analysis of the concentrated petroleum ether pigment (Fig. 3A) revealed 3 red pigment spots with R_*f*_s of 0.4, 0.6 and 0.73 for spots 1, 2 and 3, respectively (Fig. 3B). The UV–Vis absorbance spectra of each individual spot were measured (Fig. 3C). The obtained spectra all displayed the characteristic so-called “three-finger” shape at 475, 500, and 535 nm that were typical of the bacterioruberins and spirilloxanthins (C_50-_carotenoids).

**Figure 4:**
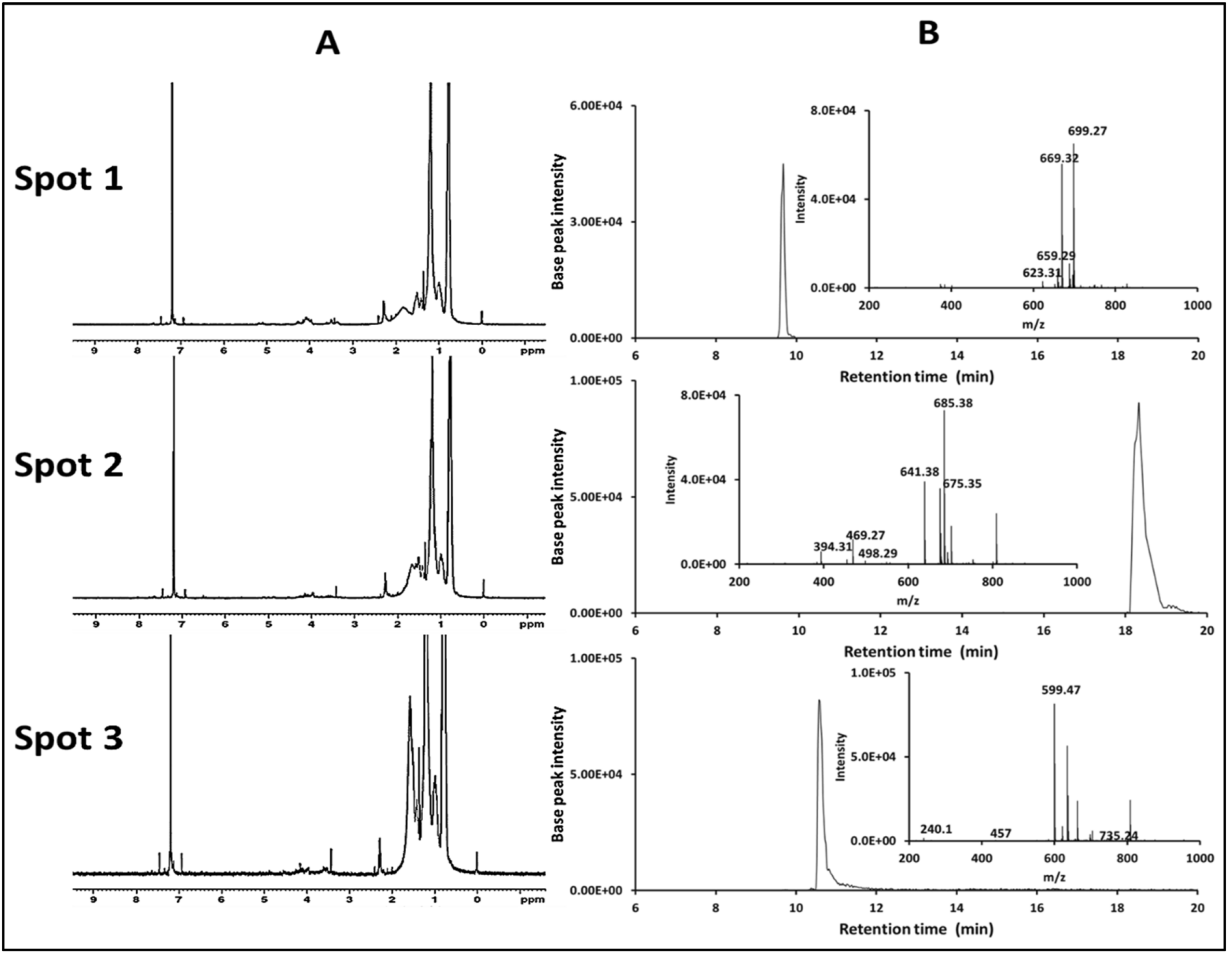
^1^H NMR spectra (**A**) and corresponding HPLC-QTOF-HRMS analysis (**B**) of the 3 *Kocuria* sp. RAM1 purified pigments (spot 1 = bisanhydrobacterioruberin, spot 2 = trisanhydrobacterioruberin and spot 3 = 3,4,3ʹ,4ʹ-tetrahydrospirilloxanthin).

The pigment derivatives were differentiated via ^1^H NMR spectra. All three pigments’ spectra revealed resonances in the 6.0–7.5 ppm range, which corresponded to different olefinic protons along the π-electron conjugated chain. Spot 1’s 1.2–1.35 ppm region represents 12 protons in 4 methyl groups next to the two hydroxyl groups (C_2_H_6_-C-OH), which can be used to distinguish it from spot 2. While in spot 2, the 1.5–2 ppm region represents the 18 protons of the 6 terminal methyl groups. In the case of spot 3, the region of 3.4–3.5 has 6 protons of the 2 methyl groups attached to the oxygen atom (–OCH_3_), and the terminal methyl group protons (12 protons) are represented in the region of 1.23–1.3 ppm. The 18 protons of methyl groups are represented in the conjugated chain in the region of 2–2.5 ppm (Fig. 4A).

HPLC-ESI-Q-TOF–MS experiments were also performed to identify the 3 compounds (Fig. 4B). The obtained retention times (RTs) of pigment spots 1, 2 and 3 were 10.59, 9.63 and 18.33 min, whose had molecular ions at m/z 699.2736 ([M − H]^−^), 685.3773 ([M − H]^−^) and 599.3518 ([M − H]^−^), respectively. From literature, spot 1 was mostly predicted as as a new bisanhydrobacterioruberin derivative (700.27 Da) calculated for C_50_H_68_O_2_ due to hydrogenation (bisanhydrobacterioruberin MW = 704 Da). Meanwhile, the 2nd spot was predicted as trisanhydrobacterioruberin (686.54 Da) with a molecular formula of C_50_H_70_O. Spot 3 was predicted as predicted as 3,4,3ʹ,4ʹ-tetrahydrospirilloxanthin (600.42 Da) with a molecular formula of C_42_H_64_O_2_.

### Statistical optimization of carotenoids

**Figure 5:**
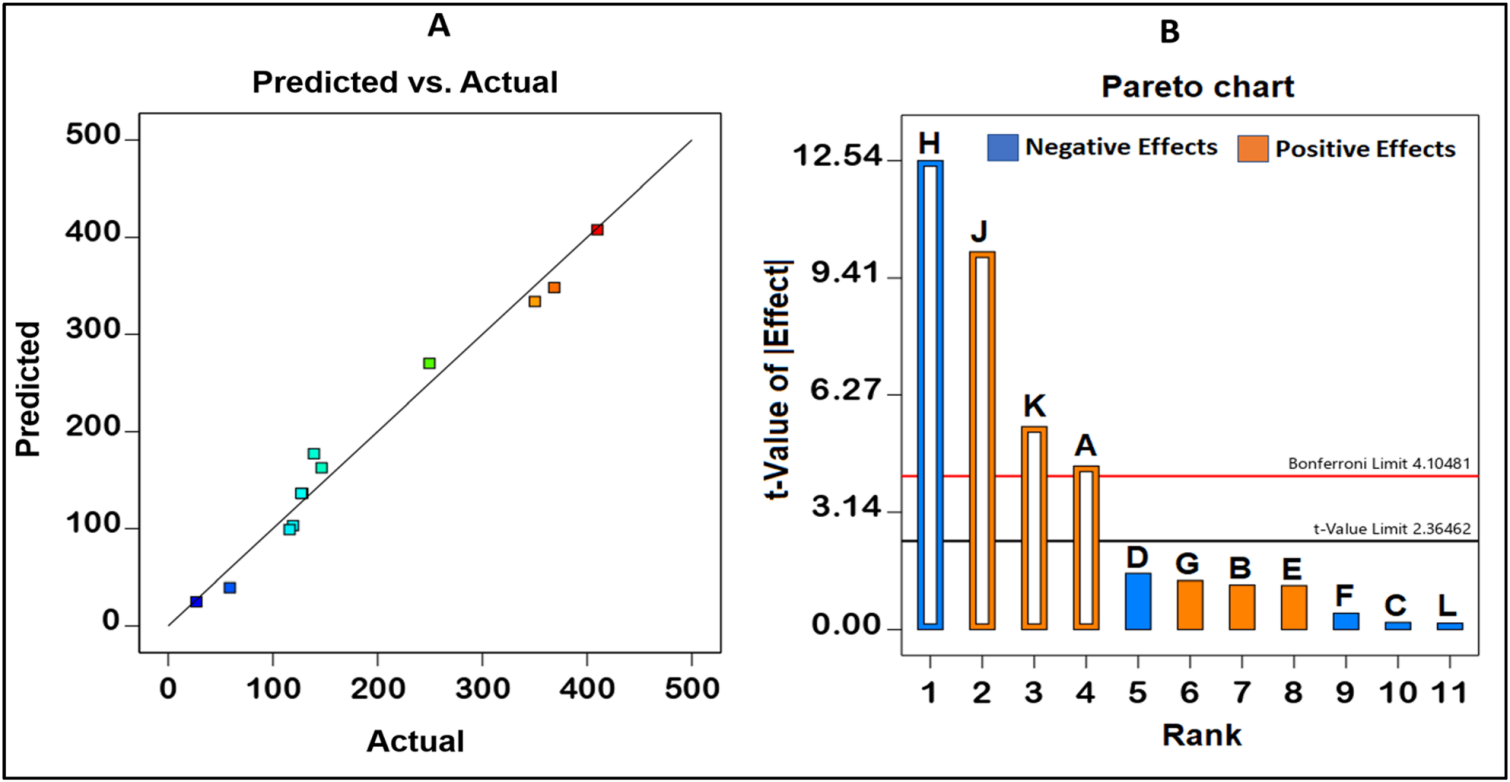
Plackett–Burman design for *Kocuria* sp. RAM1 carotenoids optimization. (**A**) Carotenoids yield predicted value versus experimental value. (**B**) PBD factors that influence carotenoids yield. The orange and blue bars are corresponding to the positive and negative effect, respectively. A: Peptone (g/l), B: Yeast extract (g/l), C: Beef extract (g/l), D: NaCl (g/l), E: Glucose (g/l), F: MgSO_4_ (g/l), G: pH, H: Temperature, J: Agitation (rpm), K: Inoculum size (%), L: Incubation period (h).

The impact of the eleven factors on carotenoid production was investigated using Plackett–Burman design (PBD) across 12 runs (Table 1). It was observed that the highest carotenoid production was seen in the 1st run (409.871 µg/ml), whereas the lowest yield was seen in 8th run (26.83 µg/ml) which was incubated at 40 °C. The model was statistically significant, as per the analysis of variance (ANOVA) (Supplementary Table S2), with a coefficient of determination (R^2^) of 0.9778, suggesting that the estimated model could explain 97.78% of the variability in the experimental data, as well as that the predicted R^2^ of 0.9347 is in sensible agreement with the R^2^ of 0.9651 (Fig. 5A). Peptone, temperature, agitation, and inoculum size were identified as significant factors (Fig. 5B) and they were considered in further optimization using response surface methodology (RSM) design. The carotenoids production model equation was as follows:

1 $${\text{Carotenoids}}\;{\text{yield}}\;\left( {\upmu {\text{g}}/{\text{ml}}} \right) = 186.62 + 29.81 {\text{Peptone }} - 85.54 {\text{Temperature }} + { 68}.{\text{92 Agitation }} + { 37}.0{\text{3 Inoculum size}}.$$

**Table 1:** Plackett–Burman design for screening the significant independent variables affecting carotenoids production by *Kocuria* sp. RAM1.

Run	Independent variables											Carotenoid (μg/ml)	
	A	B	C	D	E	F	G	H	J	K	L	Observed	Predicted
1	1	1	1	− 1	− 1	− 1	− 1	− 1	1	1	1	409.871	407.92
2	− 1	1	1	− 1	− 1	1	1	1	1	− 1	− 1	119.234	103.15
3	− 1	1	1	1	1	1	− 1	− 1	− 1	− 1	1	128.000	136.39
4	1	− 1	1	1	1	− 1	− 1	1	1	− 1	− 1	146.430	162.78
5	1	− 1	− 1	− 1	1	1	1	− 1	1	− 1	1	350.000	333.86
6	− 1	− 1	− 1	1	− 1	1	− 1	1	1	1	1	139.120	177.22
7	1	1	− 1	− 1	1	1	− 1	1	− 1	1	− 1	115.936	99.00
8	1	1	− 1	1	− 1	− 1	1	1	− 1	− 1	1	26.830	24.93
9	− 1	− 1	− 1	− 1	− 1	− 1	− 1	− 1	− 1	− 1	− 1	127.000	136.39
10	− 1	1	− 1	1	1	− 1	1	− 1	1	1	− 1	368.567	348.30
11	1	− 1	1	1	− 1	1	1	− 1	− 1	1	− 1	249.500	270.08
12	− 1	− 1	1	− 1	1	− 1	1	1	− 1	1	1	58.900	39.37

A: Peptone (g/l), B: Yeast extract (g/l), C: Beef extract (g/l), D: NaCl (g/l), E: Glucose (g/l), F: MgSO_4_ (g/l), G: pH, H: Temperature, J: Agitation (rpm), K: Inoculum size (%), L: Incubation period (h).

The response surface model (RSM) was conducted through 30 experiments (Table 2) with a 95% confidence level. The model was significant (*P*-value < 0.0001), R^2^ = 0.9270, and the predicted R^2^ (0.6600) agreed well with the adjusted R^2^ (0.8588) (Supplementary Table S3). So, ANOVA results were considered adequate for reflecting the actual relationships between the carotenoids output and the significant variables. The final empirical model in terms of a coded factor **(**A = Peptone, B = Temperature, C = Agitation, D = Inoculum size) that represents the effect of independent variables in the conducted design on carotenoids yield (µg/ml) can be expressed as follows:

2 $$\begin{aligned}{\text{Carotenoids}}\;{\text{yield}}\;\left( {\upmu {\text{g}}/{\text{ml}}} \right)& = 509.25 + 48.81 \,\,{\text{A}} - { 176.76 }\,\,{\text{B }} + { 75.91 }\,\,{\text{C }} - {23.59 }\,\,{\text{D }} - { 24.93 }\,\,{\text{A B }} \\ &\quad+ { 26}.{8}0{\text{ A C }} + { 31.39 }\,\,{\text{A D }} - { 30.63 }\,\,{\text{B C }} + { 3.76 }\,\,{\text{B D }} + \, 0.{3200}{\text{ C D }} \\ &\quad- { 43.0}{\text{9 A}}^{{2}} - { 16.06}{\text{ B}}^{{2}} + { 28.01}{\text{ C}}^{{2}} + { 19.93}{\text{ D}}^{{2}} . \end{aligned}$$

**Table 2:** The experiment design of RSM for the four independent variables affecting *Kocuria* sp. RAM1 carotenoids.

Run	Factors				Carotenoid (µg/ml)	
	A	B	C	D	Observed	Predicted
1	0	0	2	0	800.00	773.10
2	0	0	0	0	380.06	509.25
3	− 1	− 1	− 1	− 1	575.10	580.38
4	− 1	1	− 1	− 1	376.00	330.46
5	− 2	0	0	0	222.18	239.28
6	1	1	− 1	− 1	218.46	261.83
7	1	− 1	− 1	− 1	547.88	611.47
8	1	1	1	1	366.12	429.13
9	0	0	0	0	555.74	509.25
10	− 1	− 1	1	1	597.46	622.38
11	− 1	1	1	1	378.72	264.98
12	0	0	0	0	534.92	509.25
13	0	0	0	0	555.52	509.25
14	1	− 1	1	1	890.86	886.25
15	0	2	0	0	137.32	91.50
16	1	1	− 1	1	316.34	284.33
17	− 1	− 1	− 1	1	519.10	462.26
18	1	1	1	− 1	398.66	405.35
19	0	0	0	0	495.28	509.25
20	0	0	0	− 2	711.60	636.14
21	1	− 1	− 1	1	667.40	618.92
22	− 1	1	− 1	1	156.14	227.38
23	0	0	0	2	484.46	541.79
24	1	− 1	1	− 1	880.46	877.51
25	0	0	0	0	534.00	509.25
26	− 1	− 1	1	− 1	757.38	739.23
27	0	0	− 2	0	460.68	469.45
28	2	0	0	0	469.74	434.51
29	0	− 2	0	0	770.86	798.55
30	− 1	1	1	− 1	250.00	366.77

A: Peptone (g/l), B: Temperature (°C), C: Agitation (rpm), D: Inoculum size (%).

**Figure 6:**
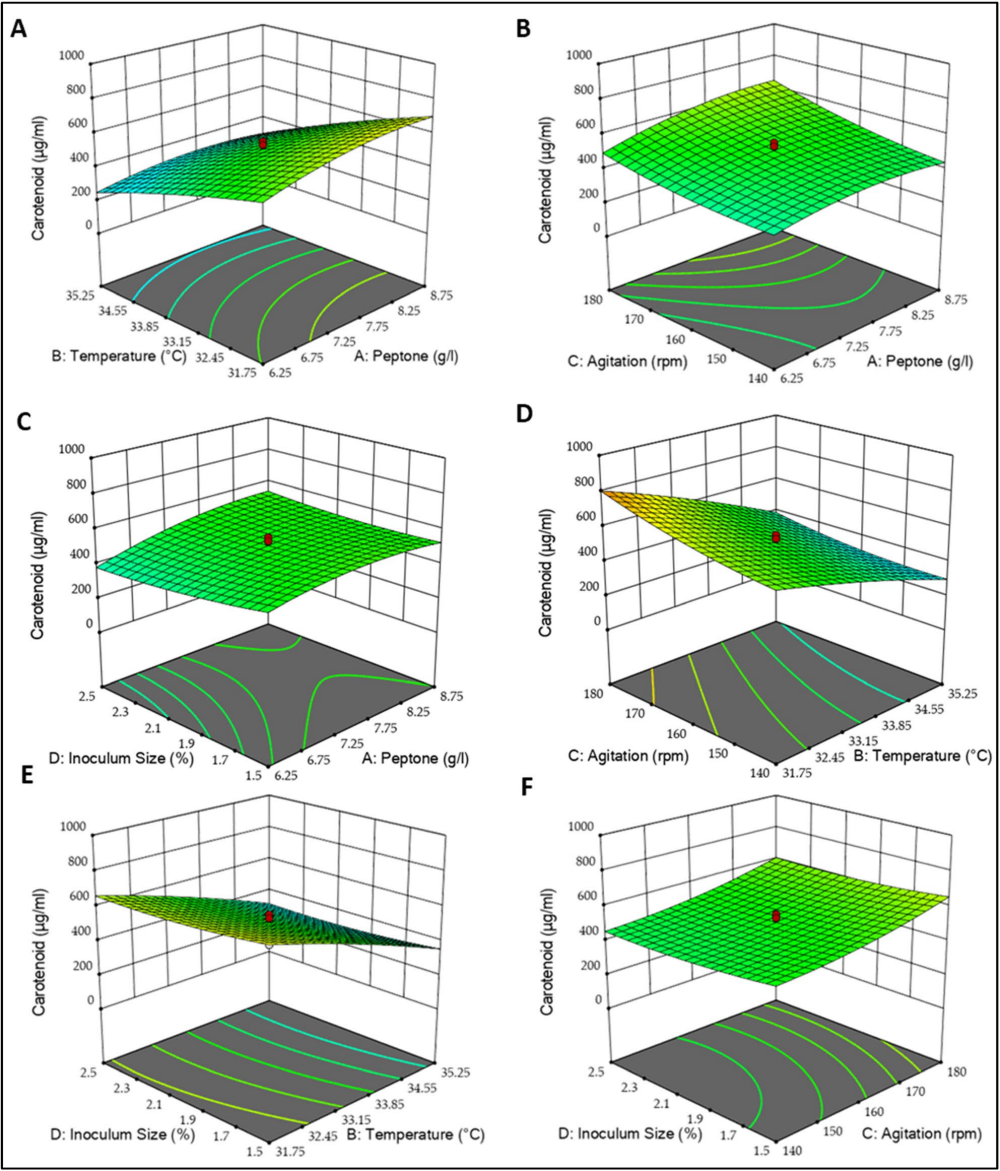
RSM plots of *Kocuria* sp. RAM1 carotenoids. (**A**) Temperature and peptone interaction, (**B**) Agitation and peptone interaction, (**C**) Inoculum size and peptone interaction, (**D**) Agitation and temperature interaction, (**E**) Inoculum size and temperature interaction, (**F**) Inoculum size and agitation interaction.

Furthermore, using three-dimensional plots, Fig. 6 demonstrates the combined effect of the four significant variables on carotenoids yield. Increases in peptone concentration at lower temperatures increased carotenoids’ yield. Further increases in temperature resulted in lower output (Fig. 6A). Higher agitation rpm with high peptone concentration resulted in 643.712 µg/ml of carotenoids, which was also observed with increasing inoculum size percentage (Fig. 6B,F). Similarly, higher inoculum size percentage also increased the output (542.417 µg/ml) (Fig. 6C). In general, the high temperature had a negative impact on the carotenoid’s synthesis (Fig. 6D,E).

The desirability function (DF) was used for carotenoids optimization. The optimal values determined by the analysis were 8.75 g/l peptone, 31.7 °C, 180 rpm, and 2.5% inoculum size, and the predicted output of carotenoids calculated using these optimal values was 886.246 µg/ml, with a desirability of 0.974. The validation resulted in a percentage error of 1.76 between the actual (870.65 µg/ml) and predicted value.

### Applications of the extracted carotenoids

#### Antibacterial activity

From MIC, it was observed that the tested Gram-negative bacteria were less vulnerable to carotenoids than the tested Gram-positive bacteria, and the results were also consistent with the zone of inhibition findings. The lack of MBC effect suggests that a concentration greater than 1000 µg/ml is required to completely kill the tested pathogens (Table 3).

**Table 3:** Minimum inhibitory concentration (MIC) and minimum bactericidal concentration (MBC) of *Kocuria* sp. RAM1 carotenoids against some bacterial pathogens.

	Pathogen	MIC		MBC
		(µg/ml)	Zone of Inhibition (mm)	(µg/ml)
Gram-positive	*Staphylococcus aureus* (ATCC 25923)	125	8 ± 1.15	1000
	*Bacillus subtilis* (ATCC 6633)	125	6 ± 0.58	> 1000
	*Enterococcus faecalis* (ATCC 29212)	500	13 ± 1.73	> 1000
Gram-negative	*Escherichia coli* (ATCC 8739)	500	10 ± 1.00	1000
	*Pseudomonas aeruginosa* (ATCC 9027)	500	11 ± 0.58	> 1000
	*Klebsiella pneumoniae* (ATCC 13883)	500	9 ± 1.73	1000

#### Anti-inflammatory activity (hypotonic solution-induced hemolysis)

**Figure 7:**
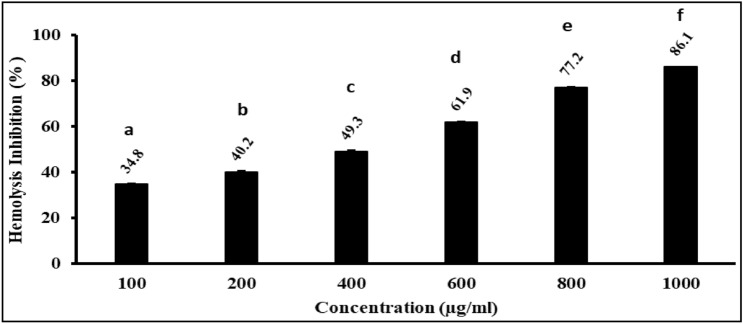
Anti-inflammatory activity of *Kocuria* sp. RAM1 carotenoids. The letters a, b, c, d, e and f represent significant differences among different homogeneous subsets identified by the post hoc test for *p* < 0.05. The data represent the mean ± SD (n = 3).

It was found that the extracted carotenoids possess concentration- dependent red blood cell (RBC) membrane stabilization, adequately protecting them against the hypotonic solution (Fig. 7). The carotenoids’ hemolysis inhibition% ranged from 34.84 ± 0.31 to 86.14 ± 0.36.

#### Antioxidant activity (DPPH radical scavenging activity)

**Figure 8:**
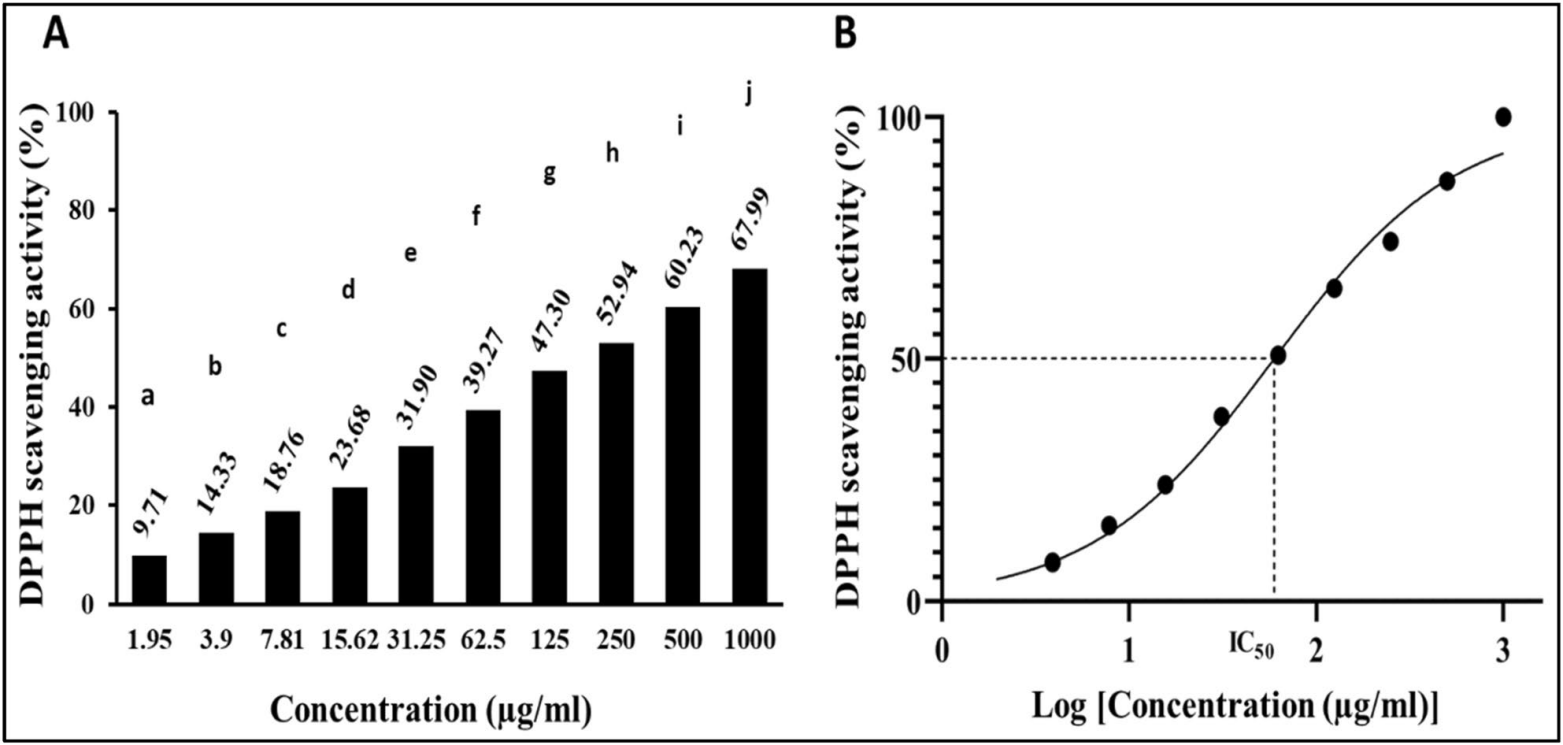
(**A**) Antioxidant activity of *Kocuria* sp. RAM1 carotenoids. The letters from a to j represent significant differences among different homogeneous subsets identified by the post hoc test for *p* < 0.05. (**B**) IC_50_ estimation. The data represent the mean ± SD (n = 3).

The main objective of the DPPH study was to assess the antioxidant activity of the extracted carotenoids. The ability to scavenge DPPH increased in tandem with the increase in concentrations (Fig. 8A). The antioxidant activity was determined to be 67.99 ± 0.21% at the highest concentration of 1000 µg/ml, and 9.71 ± 0.28% at the lowest concentration (1.95 µg/ml). 59.67 µg/ml was the estimated antioxidant concentration aimed to reduce 50% of the initial DPPH concentration (IC_50_) (Fig. 8B).

#### Maximum non-toxic dose (MNTD) of *Kocuria* sp. RAM1 carotenoids

**Figure 9:**
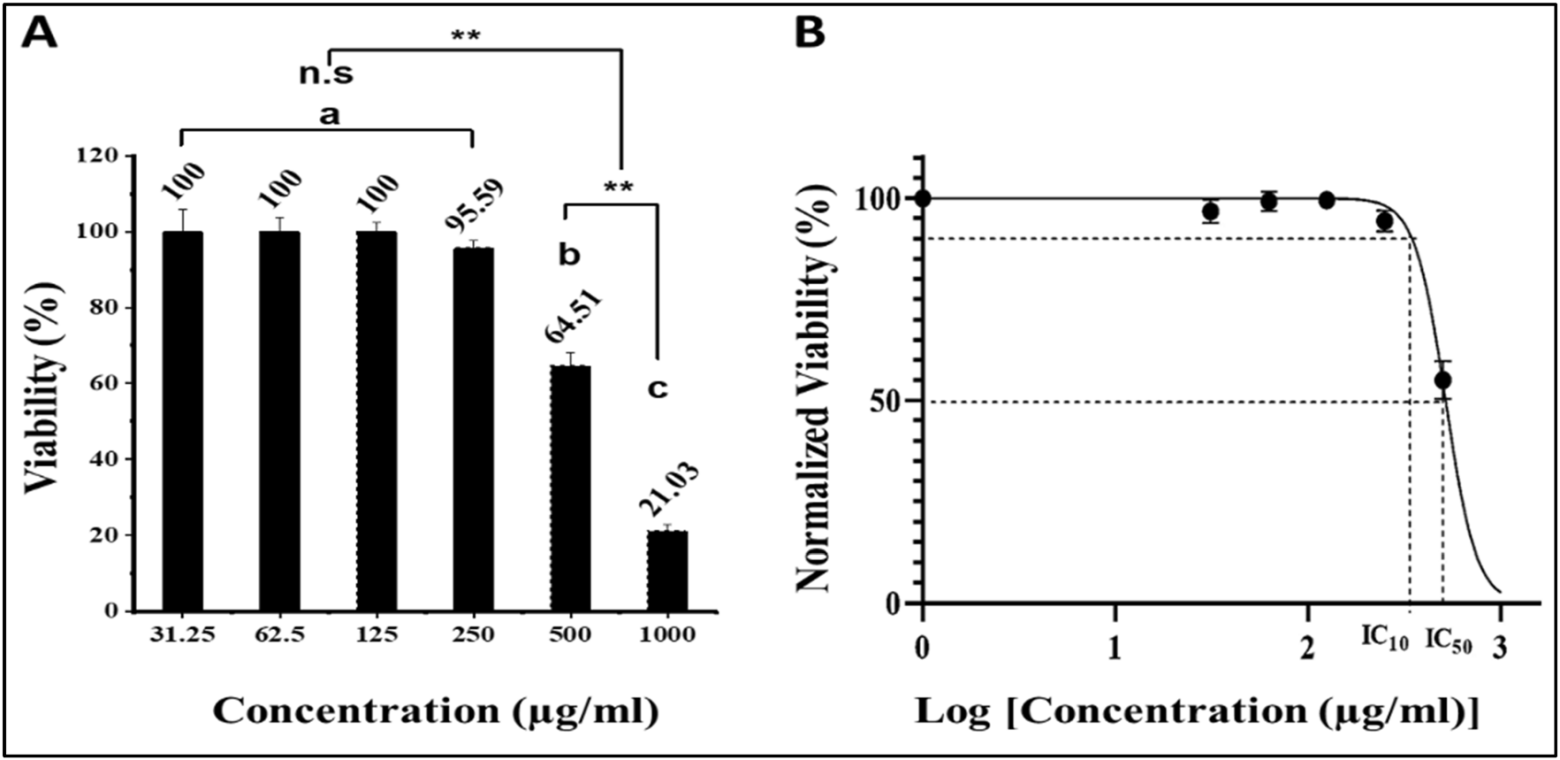
MNTD estimation of *Kocuria* sp. RAM1 carotenoids. (**A**) Cytotoxicity on normal Vero cells*.* The letters a, b and c represent significant differences among different homogeneous subsets identified by the post hoc test for *p* < 0.05. (**)Significant at *p* < 0.01; *n.s* non-significant. (**B**) IC_50_ estimation. The data represent the mean ± SD (n = 3).

We preferred the Vero cell line as a base in the in vitro model for our study due to its toxicity sensitivity, culture ease, and availability. It was observed that there was no cytotoxic effect of the extracted carotenoids at concentrations of 31.5, 62.5 and 125 µg/ml; a slight toxicity was recorded at 250 µg/ml (95.59%); and the cell viability was significantly reduced at 500 and 1000 µg/ml (Fig. 9A). The estimated IC_50_ was 516.9 µg/ml (Fig. 9B), and the MNTD for normal morphology and cell density in the presence of the extracted carotenoids was 250 µg/ml.

#### Wound healing

**Figure 10:**
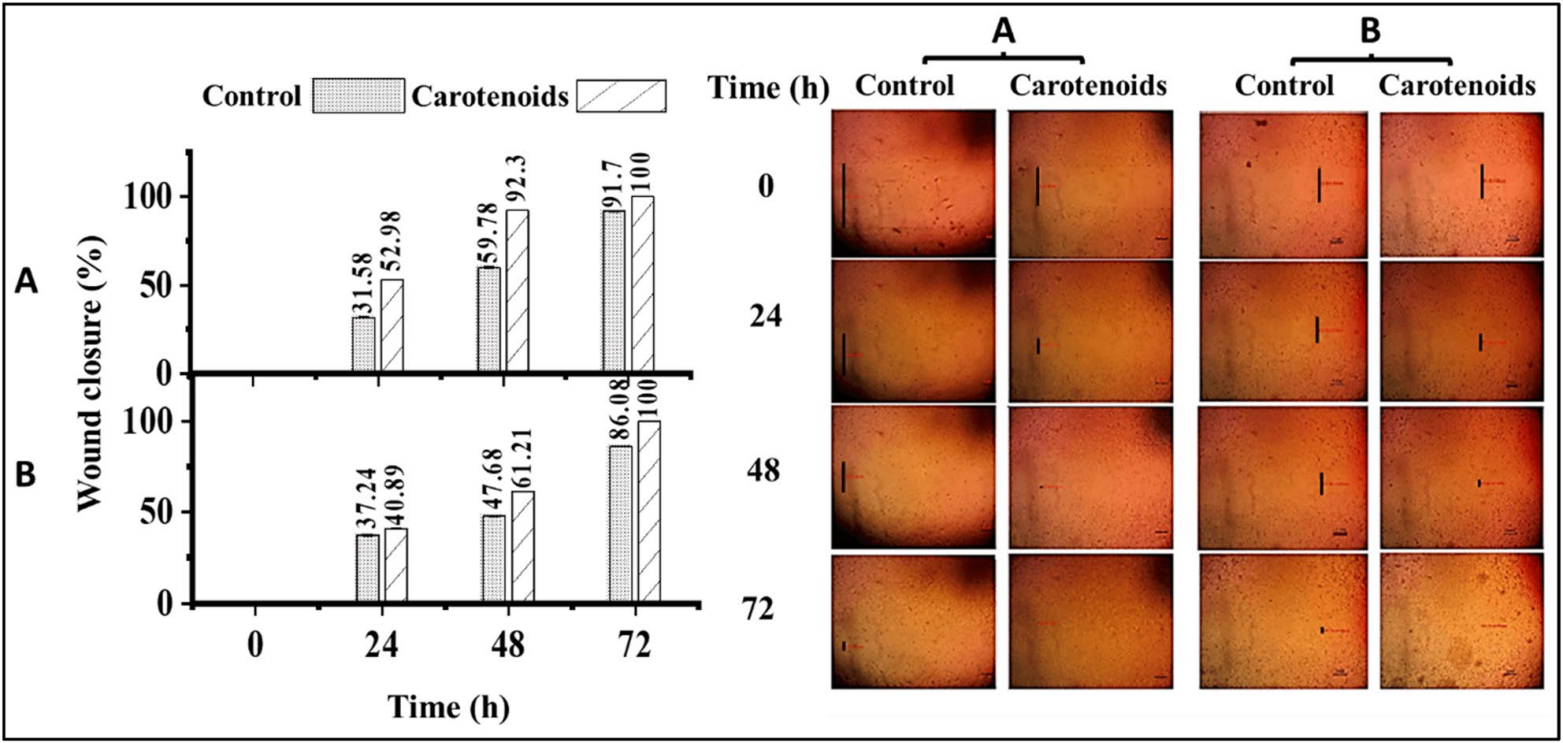
Wound closure percentage using *Kocuria* sp. RAM1 carotenoids of (**A**) human skin fibroblasts (HSF) and (**B**) oral epithelial cells (OEC) at different time intervals (0, 24, 48 and 72 h). Cells were injured by a straight-line scratch across the monolayer, and photographs were captured at different time intervals (0, 24, 48 and 72 h). The data represent the mean ± SD (n = 3).

The wound healing process of human skin fibroblasts (HSF) and oral epithelial cells (OEC) was evaluated by applying the MNTD (250 µg/ml) of the extracted carotenoids. Every 24 h, wound size reduction was observed and calculated (Fig. 10). The extracted carotenoids showed an increase in the wound healing of HSF cells. The first obtained data after 24 h revealed wound closure of 52.98% higher than that of control (31.58%). After 48 h, the wound size was reduced by 92.32% compared to the control (59.78%). After 72 h, complete recovery was observed, while the control wound was completely closed after 96 h (Fig. 10A). In the case of OEC (Fig. 10B), after 24 h, wound closure percentage values for the two groups were found to be similar, with values of 37.24 and 40.89% for control and carotenoids, respectively. The wound closure rate increased after 48 h, reaching 61.21% when compared to the control (47.68%). In the case of carotenoids, a complete recovery was observed after 72 h, whereas control wound closure did not occur (86.08%), which closed completely after 96 h. Previous results prove that the extracted carotenoids can promote wound closure activity, resulting in a faster healing process of HSF and OEC.

#### Anti-herpes simplex virus type 1 (HSV-1)

**Figure 11:**
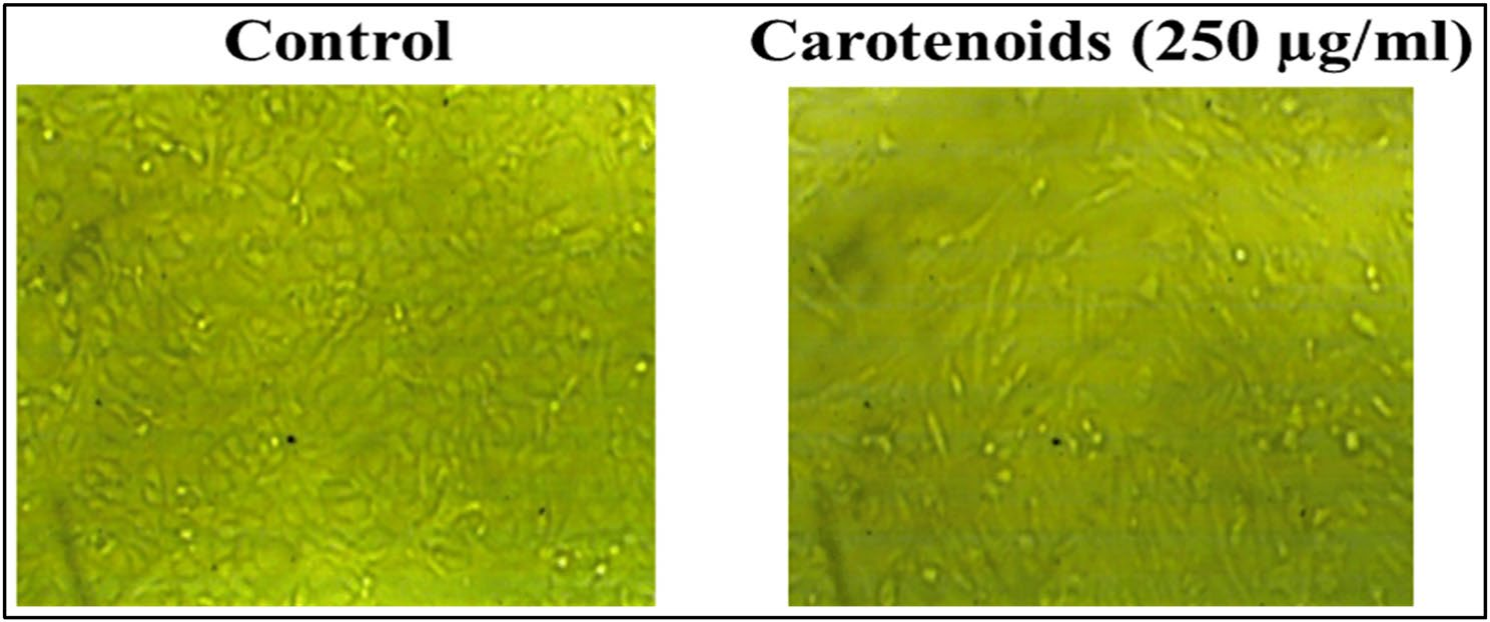
Effect of *Kocuria* sp. RAM1 carotenoids on HSV-1 infectivity. The data represent the mean ± SD (n = 3).

A moderate HSV-1 infection inhibition was estimated as 64.44% ± 5.03 (> 50% and < 90%) in Vero cells after the addition of *Kocuria* sp. RAM1 carotenoids-treated HSV-1, which is a promising outcome (Fig. 11).

#### Anticancer activity against MCF-7, Caco-2 and HeLa

**Figure 12:**
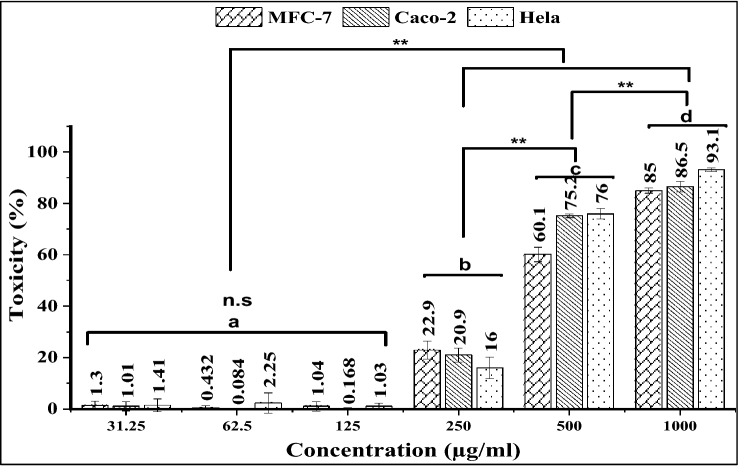
Cytotoxicity of *Kocuria* sp. RAM1 carotenoids against MCF-7, Caco-2 and Hela cell lines. The letters a, b, c and d represent significant differences among different homogeneous subsets identified by the post hoc test for *p* < 0.05. (**)Significant at *p* < 0.01; *n.s* non-significant. The data represent the mean ± SD (n = 3).

*Kocuria* sp. RAM1 carotenoids exhibited no cytotoxicity up to 125 µg/ml, indicating that they did not affect the cell viability, whereas the toxicity increased at higher concentrations of 250, 500, and 1000 µg/ml (Fig. 12). The inhibitory IC_10_, IC_50_ (µg/ml) and SI (selectivity index) were calculated using GraphPad Prism 9 (Fig. 13). The extracted carotenoids displayed a higher degree of selectivity against the Caco-2 cell line (1.6) compared to the moderate selectivity (1.4) in Hela and MCF-7 cancer cell lines (SI < 1.5). The SI for cancer cell lines was calculated by dividing the IC_50_ obtained for Vero cells (516.9 µg/ml) by the IC_50_ obtained for the cancer cells.

**Figure 13:**
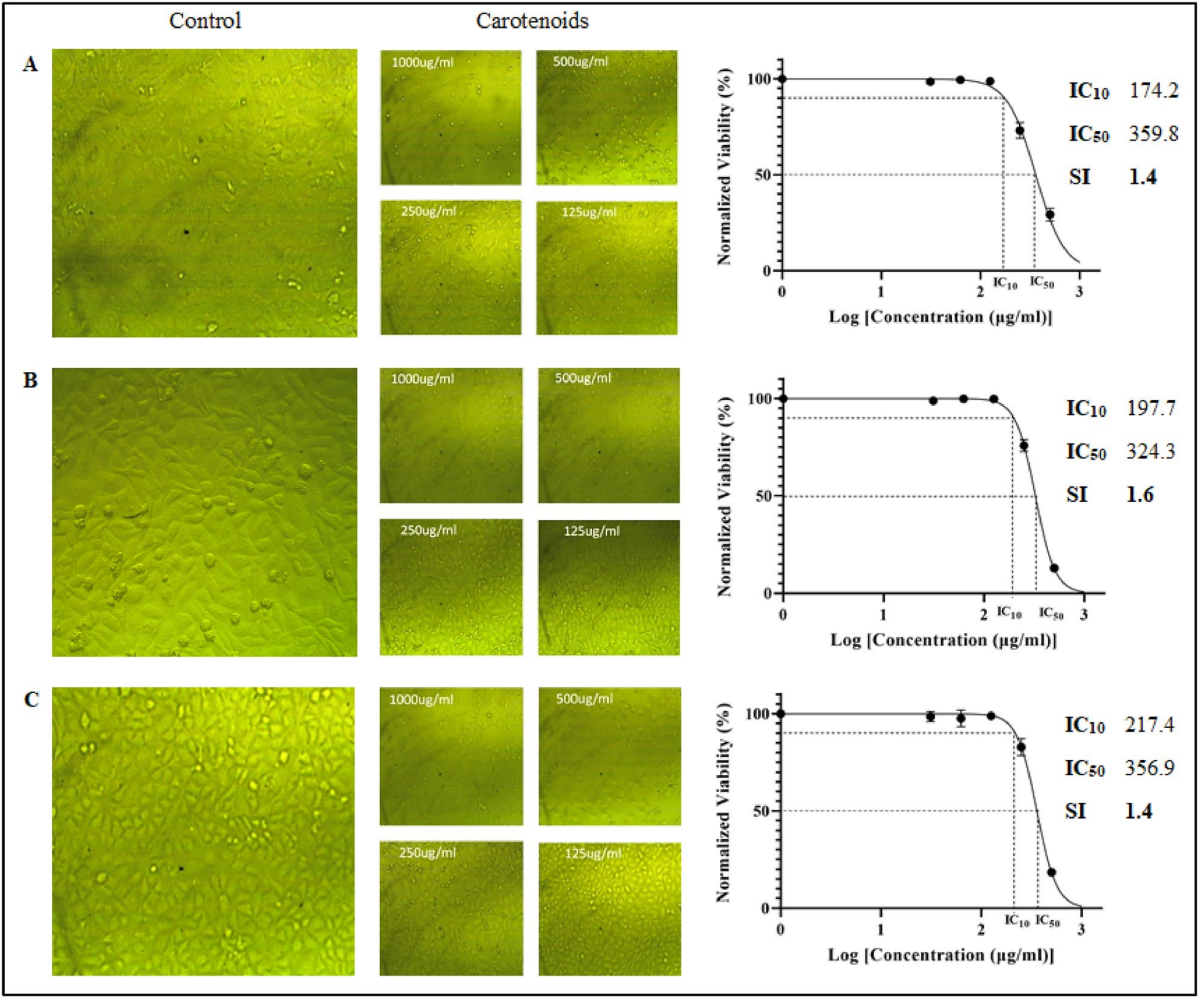
Imaging and IC_10_ and IC_50_ estimations of (**A**) MCF-7, (**B**) Caco-2 and (**C**) Hela cancer cell lines treated with different concentrations of *Kocuria* sp. RAM1 carotenoids by the in vitro cytotoxicity MTT test. GraphPad Prism 9 was used for plots.

#### Antidiabetic activity (α-glucosidase inhibition)

The in vitro antidiabetic potential is manifested by inhibiting α-glucosidase enzyme. According to the findings, the extracted carotenoids have concentration-dependent inhibitory activity for α-glucosidase (Fig. 14A). The calculated IC_50_ values were 22.77 and 35.52 µg/ml for acarbose and carotenoids, respectively (Fig. 14B). Lower IC_50_ values indicated higher inhibition. So, the previous data revealed that the extracted carotenoids seem to be slightly less potent in α-glucosidase inhibitory potential compared to acarbose (standard).

**Figure 13:**
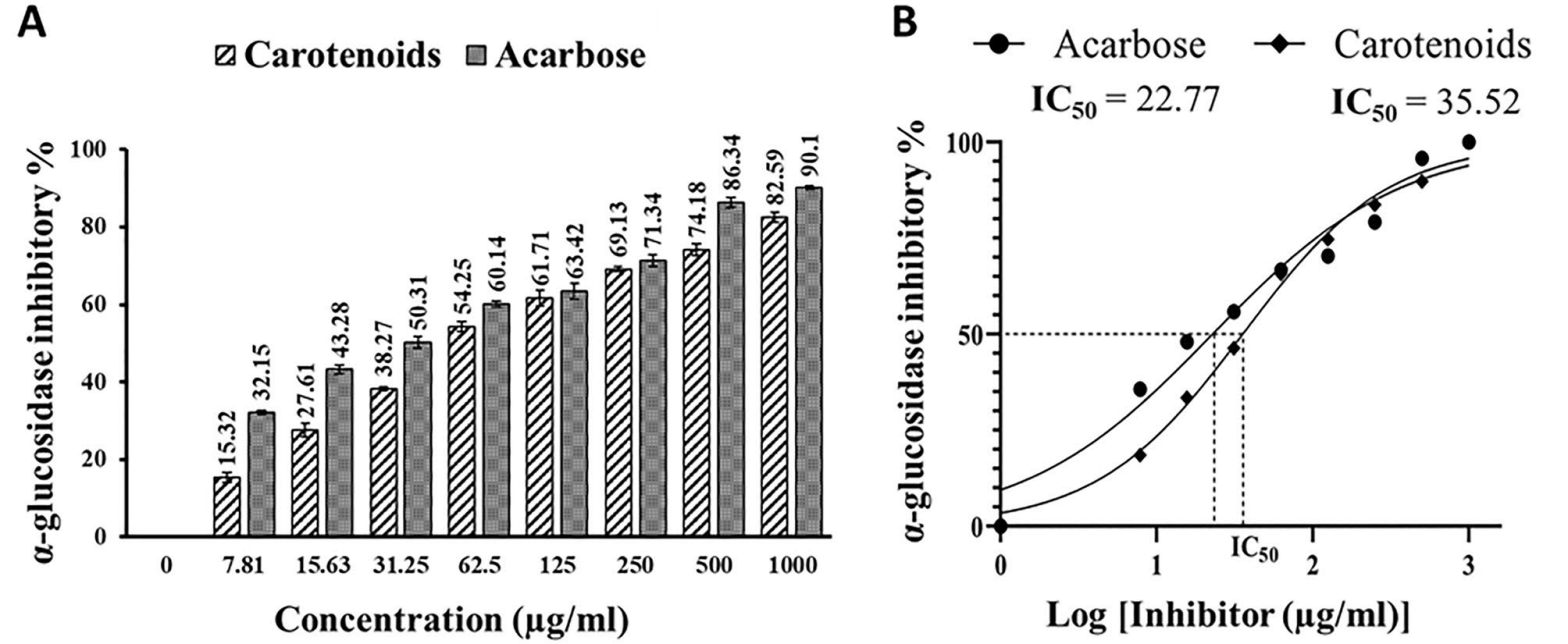
(**A**) In vitro α-glucosidase inhibitory effect of *Kocuria* sp. RAM1 carotenoids vs. acarbose (positive control) at different concentrations. (**B**) IC_50_ estimations. The data represent the mean ± SD (n = 3).

## Discussion

In this study, a marine pigmented bacterium *Kocuria* sp. RAM1 from was isolated and identified with detailed chemical and molecular characterization. From previous studies, it is known that the associated bacterial communities are generally distinct from those found in the water column, exhibiting distinct characteristics, and producing structurally and functionally novel biologically active molecules^9^.

*Kocuria* sp. RAM1 was revealed to be a good source to produce a new carotenoid mixture as a secondary metabolite dominated by C_50_-carotenoids. After extraction and purification, the pigment was characterized using TLC, UV, ^1^H-NMR and HPLC-Q-TOF–MS methods. The initial data obtained from the TLC experiment led to the recognition of 3 compounds, and UV spectra set the way to the expectation for the compounds to be bacterioruberin (C_50_-carotenoid) and spirilloxanthin derivatives because of the characteristic so-called “three-finger” shape^10–13^. By using ^1^H NMR, both spots 1 and 2 were validated as bacterioruberin derivatives that differ from the 3rd one. But it was necessary to confirm these results by finding the molecular weight. HPLC-Q-TOF–MS confirmed the molecular weight of the 3 compounds as 3,4,3ʹ,4ʹ-Tetrahydrospirilloxanthin, bisanhydrobacterioruberin derivative and trisanhydrobacterioruberin, respectively. Bacterioruberin derivatives have been found in several strains, including *Azospirillum brasilensis, Micrococcus roseus, Rubrobacter radiotolerans, Arthrobacter agilis, Kocuria rosea,* or *Thermus filiformis*^14,15^. *Kocuria* are unique sources of various pigments, including neurosporene^16^, sarcinaxanthin^17^ and β-Cryptoxanthin^18^.

Our study revealed that the incubation temperature highly influenced carotenoid synthesis. At high temperature, low carotenoids were produced due to low bacterial growth, which may be due to the denaturation of the enzyme system of microorganisms^19^. Previous studies showed that incubation of *E. coli* at 22 and 28 °C resulted in better lycopene production compared to 37 °C^20,21^, while the maximum carotenoids production by *Bacillus clausii* was achieved at 35 °C via the Taguchi method of optimization. Also, carotenoids concentration increased significantly in cultures grown at 180 rpm due to increased air supply, which increased the biomass. Peptone is an important nitrogen source that was found to be supportive of the production of biomass and carotenoids^22^. It was found that β-carotene was optimally produced by *Serratia marcescens* RB3 in the presence of 2% (w/v) peptone^23^.

Given the appealing properties of carotenoids, there has recently been a greater emphasis on investigating new natural sources of carotenoids that may greatly aid in the handling of many diseases (antibacterial, anti-inflammatory, antioxidant, wound healing, anti-HSV-1, anticancer and antidiabetic).

The potent finding in this study was the antibacterial activity of *Kocuria* sp. RAM1 carotenoids against *K. pneumoniae*, which is well-known for evolving multidrug resistance, and was reported in COVID-19 intensive care unit (ICU) patients who may be affected by bacterial superinfection, causing an increase in the fatality rate^24–26^. The other finding was the antibacterial activity against *S. aureus, P. aeruginosa* and *E. coli* that mostly cause wound infections, particularly in hospitals due to antibiotic resistance mechanisms that are easily acquired^27,28^, giving us an incentive to test it in a wound healing process after determination of the MNTD, which turned out to be equal to 250 µg/ml. And regarding this application, it was reported that bacterioruberin carotenoids extracted from *Aquisalibacillus elongatus* MB592, *Salinicoccus sesuvii* MB597, and *Halomonas aquamarina* MB598 revealed antibacterial activity against *B. subtilis, B. pumilus, Enterococcus faecalis, B. cereus, Alcaligenes faecalis, Klebsiella pneumoniae, Pseudomonas geniculata, and Enterococcus faecium,* with relatively less inhibition observed against *B. subtilis*^29^. This is in addition to different bacterioruberin derivatives that were extracted from haloarchaea strains and showed antibacterial activity against *E. coli, K. pneumonia, P. aeruginosa and S. aureus*^30^*.*

Finding a natural alternative to drugs like NSAIDs (nonsteroidal anti-inflammatory drugs) that have negative side effects and should be avoided by people with certain medical problems is beneficial^31,32^. RBCs were used for inflammation inhibition evaluation of *Kocuria* sp. RAM1 carotenoids as their membrane behaves similarly to the lysosomal membrane. When stabilized by carotenoids, the lysosomal membrane is also stabilized or protected, resulting in inflammation inhibition^33,34^. Our extracted carotenoids showed a significant potent on human erythrocytes, adequately protecting them against the hypotonic solution. The previously reported results of the in vitro hemolysis assays lack comparability and contain numerous variations due to experiment settings primarily related to the concentration of the used carotenoids and the density of the erythrocyte suspension. Some microbial carotenoids, including β-carotene, lycopene, lutein, astaxanthin, zeaxanthin and keto-myxocoxanthin glucoside-ester, exhibited anti-inflammatory activities^35,36^. In another study, β-cryptoxanthin had no erythroprotective effect but instead caused hemolysis at the highest tested concentration^37^_._

Carotenoids in general, particularly those from marine origins, are excellent antioxidants^38–40^. This is attributed to structural properties such as the conjugated double-bond length and the existing chemical groups^41–44^. In our study, two of the identified pigments were C_50_-carotenoids, characterized by 13 conjugated double bonds and the presence of a hydroxyl group^45^. According to previous studies, bacterioruberin, bisanhydrobacterioruberin, trisanhydrobacterioruberin, and their derivatives have been shown to have significant antioxidant capacity, possibly greater than β-carotene^46,47^. In addition, it has been proven that spirilloxanthin extracted from *Bacillus licheniformis* Rt4M10 has also an antioxidant scavenging capacity^48^.

Our study supported the use of *Kocuria* sp. RAM1 carotenoids as a promising option or additive in the wound healing process of both HSF and OEC. The ultimate goal of wound healing is to have a speedy recovery, which was accomplished faster than the control cell lines in our study. This is a great finding in addition to the previously mentioned antimicrobial, antioxidant, and anti-inflammatory behaviors that are required during the defensive phase of inflammation^49^. It was reported that carotenoids such as zeaxanthin, pyranone and astaxanthin led to faster healing in comparison with control in both in vitro and in vivo studies^50–53^. Our results may encourage further in vivo and clinical investigations to elucidate the effects of the wound healing process.

HSV-1 and HSV-2 are two of the main factors that cause oral-labial, genital herpes, blindness and a high mortality rate in encephalitis^54–56^. The overuse of anti-HSV drugs has given rise to drug-resistant viral strains, which creates an urgent need for new anti-HSV agents^57^. The present results hint that *Kocuria* sp. RAM1 carotenoids may intrude into the early stages of the viral infectious cycle or replication, such as virus binding and penetration, by obstructing virus-host cell attachment, or it may suggest that the pigment diminishes susceptibility to infection by trying to target host factors that enhance viral replication. The current study demonstrates for the first time that such extracted carotenoids can be tested as anti-HSV-1. Most studies are related to algal carotenoids, as it was reported that crocin and picrocrocin (a derivative of zeaxanthin) had significant in vitro anti-HSV-1 activity through inhibition of HSV replication before and after entry into Vero^58^, in addition to *Dunaliella* *salina* and *Haematococcus pluvialis* carotenoids that reduced its activity ranging from 50 to 85%^59^. Some bacterial pigments rather than carotenoids, such as prodigiosin, exert antiviral activity against HSV-1 and HSV-2 infections^60^ in comparison to violacein, which showed weak inhibition^61^. Therefore, our findings suggest that *Kocuria* sp. RAM1 carotenoids could be a new class of antiviral compounds, although more research is required to explore the involved antiviral mechanism.

As cancer is the leading cause of death worldwide, many studies have been conducted in recent years on cancer therapy using natural products. *Kocuria* sp. RAM1 carotenoids showed selective cytotoxicity (SI > 1) on MCF-7, Caco-2 and Hela cancer cells, indicating their anticancer activity, but more research is needed to pinpoint their precise mechanism of action and determine the best clinical application. It was reported that β-carotene and canthaxanthin have a selective cytotoxic effect on different tumor cell lines, including oral carcinoma, breast, lung carcinoma, and malignant melanoma^62^, as well as β-cryptoxanthin which decreased the proliferation of colon cancer cells^63^. Also, several types of carotenoids showed anticancer activity against human leukemia (HL-60), breast (MCF-7) and colon (Caco-2) cancer cells^64–68^. Some case studies supported the notion that a high dietary intake of carotenoids was linked to a lower risk of colorectal and breast cancer^69,70^.

Finally, the current study provides evidence to support the use of *Kocuria* sp. RAM1 carotenoids as an anti-diabetic agent, or as a nutraceutical or potential source of diabetes drugs. Similarly, it has been proven that carotenoids such as lutein (IC_50_ = 70 µmol/l), zeaxanthin (IC_50_ = 53.5 µmol/l) and fucoxanthin (IC_50_ = 4.75 mmol/l) exhibited α-glucosidase inhibition activity^71,72^. In addition, some studies have revealed that consuming carotenoids (β-carotene, α-carotene, β-cryptoxanthin, lycopene, lutein and zeaxanthin) reduces type 2 diabetes risk in men and women^73,74^.

## Materials and methods

### Isolation and selection of pigmented bacteria

Invertebrate samples were collected from Marsa Alam, Red Sea, Egypt. The selected samples represented different marine invertebrates of the Echinodermata phylum. The samples were rinsed with sterile seawater, cut with a scalpel aseptically, homogenized using a sterile mortar and pestle, and then transferred to sterile bottles. One milliliter of homogenate was transferred into 50 ml of sterile nutrient broth prepared with seawater (pH 7 ± 0.2), then incubated at 30 °C for 24 h under shaking conditions (120 rpm) before transferring to agar plates for 72 h^75^. After incubation, colored colonies were picked, purified, sub-cultured, and maintained as stock cultures for further studies.

### Biochemical and molecular identification

Biochemical characterization of the pigmented isolate was implemented via VITEK 2 (bioMérieux, Marcy l’Etoile, France)^76^. Molecular identification was carried out using the universal bacterial primers to amplify ~ 1500 bp fragment of the 16S rDNA region via polymerase chain reaction (PCR)^77^. The amplified PCR product was sequenced^78^, and the BLAST program was used to investigate similarity^79^. A phylogenetic tree was generated by MEGA version 11.0.10^80,81^.

### Pigment extraction and purification

Fifty ml of the orange-colored culture were centrifuged at 6000 rpm for 20 min after 48 h of incubation, followed by washing the pellet with distilled water and centrifuging again. Different solvents such as methanol, ethanol, acetone, and ethyl acetate were tested for the most efficient pigment extraction. The washed pellet was mixed with 50 ml of methanol and then immersed in a water bath at 40 °C for 15 min. The process was repeated until complete pigment extraction was achieved. The pigment extract was evaporated at 40 °C by a rotary evaporator to dryness^82^. The extract was dissolved in petroleum ether and mixed with methanol in a separating funnel, and vigorously shaken for 10–15 min to allow the two liquids to separate. Then, a concentrated solution of NaCl in distilled water was added to the separating funnel to improve phase separation. The pigment was collected, washed five times with distilled water to remove methanol residues, and finally concentrated using a rotary evaporator at 35 °C^83^.

### Identification of the extracted pigment

#### Thin-layer chromatography (TLC) and UV spectra

Petroleum ether pigment fraction was analyzed using thin-layer chromatography with silica gel (TLC cards, Sigma, Germany) via solvent system of chloroform: methanol (6:1; v/v). The retention factor (R*f*) was estimated as follows:

3 $$\text{R}f=\frac{\text{Distance travelled by the compound}}{\text{Distance travelled by the solvent front}}.$$

Each spot having an identical R*f* value was studied as a single compound. Such spots were scraped off, accumulated, and the solvent was evaporated to yield the high purity dried pigment before being dissolved and scanned at 400–600 nm to obtain the UV–Vis absorbance spectrum of each individual spot^84^.

#### Nuclear magnetic resonance (NMR) spectroscopy

The purified compounds were characterized by ^1^H nuclear magnetic resonance (^1^H-NMR) (JEOL ECZ 500) using deuterated chloroform (CDCl_3_) as a solvent at frequency of 500 MHz^85^.

#### HPLC-ESI-Q-TOF–MS

The three pigment spots were identified via HPLC-ESI-QTOF-MS (X500R LC-QTO, SCIEX, USA) in negative ionization mode. Mobile phase was made up of acetonitrile/tetrahydrofuran/methanol (58:7:35) at a flow rate of 2 ml/min^86^. The mass spectrometry conditions were performed with a scanning range of 100–1500 m/z, a capillary voltage of 3.2 kV, a cone voltage of 40 eV, and a source temperature of 120 °C. Nitrogen was used as the nebulizing gas at a flow rate of 30 l/h. The MS/MS spectra were measured with argon as a collision gas (collision energy = 30 V).

### Statistical optimization of carotenoids

First, Plackett–Burman experimental design (PBD) was performed to identify the crucial variables affecting carotenoid production as a response using Design Expert (Version 11 Stat-Ease Inc., Minneapolis, MN, USA) (Table 4)^87^. The used first-order polynomial equation was as follows^88^:

4 $$\text{Y}={\beta }_{0}+\sum \limits_{i=1 }^{K}{\beta }_{i }Xi \left(i=1, \dots ., K\right),$$

where Y is the response (carotenoids yield in µg/ml), *β*_0_ is the model intercept, β_*i *_is a linear regression coefficient, X*i* is the level of independent variables and K is the number of variables.

**Table 4:** Experimental variables used in PBD design for screening of *Kocuria* sp. RAM1 carotenoids production.

Independent variables	Symbol	Unit	Experimental value	
			Low (− 1)	High (+ 1)
Peptone	A	g/l	1.0	9.0
Yeast extract	B	g/l	1.0	3.0
Beef extract	C	g/l	1.0	5.0
NaCl	D	g/l	0.0	10.0
Glucose	E	g/l	0.5	1.5
MgSO_4_	F	g/l	0.5	1.5
pH	G	–	4.0	10.0
Temperature	H	°C	30.0	40.0
Agitation	J	rpm	90.0	150.0
Inoculum size	K	%	0.5	1.5
Incubation period	L	h	24.0	72.0

The four significant PBD variables, peptone (g/l), temperature (°C), agitation (rpm) and inoculum size (%), were evaluated as factors, and carotenoid yield was recorded as the response in the central composite inscribed design (CCI) using Design Expert (Table 5)^89^. The relationship between the independent factors and dependent responses was calculated by using a quadratic polynomial equation as follows^90^:

5 $$\text{Y}={X}_{0}+\sum \limits_{i=1}^{\text{K}}{\beta }_{i}{X}_{i} +\sum \limits_{i=1}^{\text{K}}{\beta }_{ii}{X}_{i}^{2}+\sum \sum \limits_{i< j=1}^{\text{K}}{\beta }_{ij}{X}_{i} {X}_{j}+\text{E},$$

where Y is the response (carotenoids yield in µg/ml), X_0_ is the intercept or regression coefficient; *β*_i_, *β*_ii_, and *β*_ij_ are the linear, quadratic and interaction coefficients respectively; X*i* and X*j* are the coded values of the variables; E is the experimental/residual error and K is the number of variables.

**Table 5:** Experimental ranges of the most significant independent variables used in response surface method (RSM).

Name	Code	Units	Variable level codes				
			− α	− 1	0	+ 1	+ α
Peptone	A	g/l	5	6.25	7.5	8.75	10
Temperature	B	°C	30	31.75	33.5	35.25	37
Agitation	C	rpm	120	140.00	160.0	180.00	200
Inoculum size	D	%	1	1.50	2.0	2.50	3

### Applications of *Kocuria* sp. RAM1 carotenoids

#### Antibacterial activity

The minimum inhibitory concentration (MIC) and the minimum bactericidal concentration (MBC) of *Kocuria* sp. RAM1 carotenoids were evaluated for a total of 6 bacterial strains, including Gram-positive and Gram-negative by using the broth dilution method in a 96-well plate^91^. The minimum concentration of carotenoids that did not allow the growth of bacterial strains was recorded as MIC. MBC values were investigated by streaking onto Mueller Hinton Agar (MHA) and for 24 h. Following the incubation period, the concentration at which no visible growth was recorded as MBC. Additionally, the agar-well diffusion method was used for a zone of inhibition determination^92^.

#### Anti-inflammatory activity (hypotonic solution-induced hemolysis)

The anti-inflammatory activity of the extracted carotenoids was assessed using the human red blood cells (RBCs) membrane stabilization method via hypotonic solution-induced hemolysis^93^. Serial concentrations of carotenoids ranging from 100 to 1000 µg/ml were mixed with erythrocyte suspension, incubated for 1 h at room temperature, and then centrifuged for 5 min at 2500 rpm. The membrane rupture of RBCs in a hypotonic solution was compared to that of an isotonic one. Instead of carotenoids, PBS and distilled water were served as negative and positive controls, respectively. The supernatants’ absorbance (OD) was measured at 540 nm, from which the hemolysis inhibition% was calculated as follows:

6 $$\text{Hemolysis inhibition} \; \%=\left[1-\left(\frac{\text{OD}2-\text{OD}1}{\text{OD}3-\text{OD}1}\right)\right]\times 100,$$

where OD1 = absorbance of test sample in isotonic solution, OD2 = absorbance of test sample in hypotonic solution (distilled water), OD3 = absorbance of control sample in hypotonic solution (distilled water).

#### Antioxidant activity (DPPH radical scavenging activity)

In brief, 100 µl of 0.1 mM of freshly prepared DPPH reagent were mixed with serially diluted concentrations of the extracted carotenoids (100 µl). Upon 30 min of dark incubation at room temperature, the DPPH color intensity was measured at 540 nm^94^. The percentage of inhibition of DPPH oxidation was calculated as follows:

7 $$\text{DPPH Scavenging activity}\; \left(\%\right)=\left(\frac{{\text{Abs}}_{\left(\text{Control}\right)}-{\text{Abs}}_{\left(\text{Sample}\right)}}{{\text{Abs}}_{\left(\text{Control}\right)}}\right)\times 100.$$

#### Determination of maximum non-toxic dose (MNTD) by MTT asssay

The Vero cell line (African green monkey kidney cell line) was used as an appropriate cell line via the MTT assay^95^. The cells (10^4^/well) were cultured in a 96-well plate (37 °C, 5% CO_2_). Carotenoids were subjected to in vitro cytotoxicity testing to determine the maximum non-toxic dose (MNTD) to Vero cells at various concentrations. 20 µl of MTT [3-(4,5-dimethylthiazol-2-yl)-2,5-diphenyltetrazolium bromide] were transferred into each well and incubated at 37 °C for 1–4 h. The absorbance was measured at 570 nm after mixing with 100 µl of the solubilization solution (DMSO). The existence of viable cells was marked by the appearance of a purple color caused by formazan crystals formation. Cell viability was determined in comparison to untreated control cells as a percentage. Cells in the medium represented the negative control. Concentration required to reduce 50% cell viability (IC_50_) was estimated. The maximum non-toxic dose (MNTD) was used for further biological studies (wound healing assay and antiviral activity).

#### Wound healing assay (in vitro scratch assay)

Wound healing of human skin fibroblasts (HSF) and oral epithelial cells (OEC) was investigated using in vitro scratch assay^96^. Cells were plated onto a coated 6-well plate at density of 3 × 10^5^/well and cultured in 5% FBS-DMEM for 12 h (37 °C, 5% CO_2_). After that, a horizontal scratch “wound” was introduced in the confluent monolayer, creating a gap. Negative-control wells were refilled with fresh media, meanwhile test wells were filled with media containing the carotenoids MNTD (250 µg/ml). Images of wounds were immediately captured after scratching (t = 0 h) and every 24 h (t = Δh) using an inverted microscope and figured out by MII ImageView software version 3.7. The rate of cell migration can be described as a percentage of wound closure as shown below^97^.

8 $$\text{Wound closure} \; \%=\frac{{\text{A}}_{t=0h}-{\text{A}}_{t=\Delta h}}{{\text{A}}_{t=0h}}\times 100,$$

where A_*t* =0 h_ is the initial wound area after scratching, A_*t* =∆h_ is the wound area measured after (h) hours.

#### Anti HSV-1 activity by viral inhibition in Vero cells

HSV-1 was pre-incubated with carotenoids MNTD (250 µg/ml) for 1 h (37 °C and 5% CO_2_) to assess antiviral activity. After that, the mixture (1:1) was added to the Vero culture followed by incubation for 24 h to allow the virus to take effect. Cells incubated in a virus-free medium served as a negative control. The cells were analyzed by the MTT assay as described previously. The percentages of antiviral activity were calculated by comparing HSV-1 positive cells in treated and untreated wells related to viability percentage^60,98^.

#### Anticancer activity of against MCF-7, Caco-2 and HeLa

The cytotoxicity of the carotenoids against MCF-7 (human breast cancer cell line), Caco-2 (homo sapiens colon) and HeLa (human epithelial adenocarcinoma cervix cell line) was evaluated using an MTT assay as described previously. IC_50_ was calculated, and all assays were done in triplicate**.** The selectivity index (SI) was estimated by the ratio between the IC_50_s of carotenoids on the normal Vero cells and cancerous cell lines^99^.

#### Antidiabetic activity (α-glucosidase inhibition)

Twenty microliters of the extracted carotenoids in various concentrations were mixed with 100 mM phosphate buffer (50 µl, pH 6.8) and α-glucosidase (10 µl, 1 U/ml) in a 96-well plate, then incubated at 37 °C for 15 min. Then, 5 mM p-nitrophenol-glucopyranoside (P-NPG) (20 µl) was added as a substrate to start the reaction and incubation again for 20 min. The response was terminated after the addition of Na_2_CO_3_ (50 µl, 0.1 M), followed by measuring at 405 nm. Acarbose served as a reference^100^. α-Glycosidase inhibitory activity was expressed as follows:

9 $$\text{Inhibitory activity }\left({\%}\right)=\left(\frac{{\text{OD}}_{Control}-{\text{OD}}_{Sample}}{{\text{OD}}_{Control}}\right)\times 100.$$

### Statistical analysis

The experiments were conducted in triplicates, and the results were presented as the mean ± SD. SPSS software Version 22.0 was used. Analysis of variance (ANOVA) and Tukey’s test were executed with the level of significance set at *p* = 0.05.

## Conclusion

Our study has been highlighted the use of new carotenoids mixture extracted from a newly isolated marine *Kocuria* sp. RAM1 (OL904955) in many biological activities (antimicrobial, anti-inflammatory, antioxidant, wound healing, anti-HSV-1, anticancer and antidiabetic). The obtained results could serve as a starting point for additional research and mechanism comprehension.

## Supplementary Information

Supplementary Tables.

## Data Availability

All data generated or analyzed during this study are included in this published article (and its Supplementary Information file). The datasets of DNA sequence of the isolated bacterial strain analyzed during the current study are available in the GenBank repository (Accession Number: OL904955), https://www.ncbi.nlm.nih.gov/nuccore/OL904955.1/.
