# Supplementary material for: Optimization and multiple in vitro activity potentials of carotenoids from marine Kocuria sp. RAM1
Source: Sci Rep. 2022 Oct 28;12:18203. doi: 10.1038/s41598-022-22897-4 (PMC9616409; doi:10.1038/s41598-022-22897-4)
Supplement: Supplementary file 1 — Supplementary Tables. [file 41598_2022_22897_MOESM1_ESM.docx]

**Supporting Information**

**Optimization and multiple *in vitro* activity potentials of carotenoids from marine *Kocuria* sp. RAM1**

**Rasha A. Metwally*^1^, Nermeen A. El-Sersy^1^, Amany El Sikaily^2^, Soraya A. Sabry^3^, Hanan A. Ghozlan^3^**

# *****[rashaabdou2012@hotmail.com](mailto:rashaabdou2012@hotmail.com)

**Table S 1**. Biochemical tests included in the VITEK 2 GP card for Kocuria sp. RAM1.

| **Test** | **Abbreviation** | **Result** |
| --- | --- | --- |
| D-Amygdalin | AMY | ̲ |
| Phosphatidylinositol phospholipase C | PIPLC | ̲ |
| D-Xylose | dXYL | ̲ |
| Arginine dihydrolase 1 | ADH1 | ̲ |
| Beta-galactosidase | BGAL | ̲ |
| Alpha-glucosidase | AGLU | (+) |
| Ala-Phe-Pro Arylamidase | APPA | ̲ |
| Cyclodextrin | CDEX | ̲ |
| L-aspartate arylamidase | AspA | ̲ |
| Beta-galactopyranosidase | BGAR | ̲ |
| alpha-mannosidase | AMAN | ̲ |
| Phosphatase | PHOS | ̲ |
| Leucine arylamidase | LeuA | (+) |
| L-Proline arylamidase | ProA | ̲ |
| Beta-glucaronidase | BGURr | ̲ |
| alpha-galactosidase | AGAL | + |
| L-pyrrolidonyl-arylamidase | PyrA | ̲ |
| Beta-glucaronidase | BGUR | ̲ |
| Alanine arylamidase | AlaA | ̲ |
| Tyrosine arylamidase | TyrA | ̲ |
| D-sorbitol | dSOR | ̲ |
| Urease | URE | ̲ |
| Polymixin B resistance | POLYB | ̲ |
| D-galactose | dGAL | ̲ |
| D-ribose | dRIB | ̲ |
| L-lactate alkalinization | ILATk | ̲ |
| Lactose | LAC | ̲ |
| N-acetyl-D-glucosamine | NAG | ̲ |
| D-maltose | dMAL | ̲ |
| Bacitracin resistance | BACI | ̲ |
| Novobiocin resistance | NOVO | ̲ |
| Growth in 6.5% NaCl | NC6.5 | ̲ |
| D-mannitol | dMAN | ̲ |
| D-mannose | dMNE | ̲ |
| Methyl-B-D-glucopyranoside | MBdG | ̲ |
| Pullulan | PUL | ̲ |
| D-raffinose | dRAF | ̲ |
| O/129 resistance (comp.vibrio.) | O129R | ̲ |
| Salicin | SAL | ̲ |
| Saccharose/sucrose | SAC | ̲ |
| D-trehalose | dTRE | ̲ |
| Arginine dihydrolase 2 | ADH2s | ̲ |
| Optochin resistance | OPTO | ̲ |

**Table S 2***.* Analysis of variance (ANOVA) of *Kocuria* sp. RAM1 carotenoids using Plackett-Burman design.

| **Source** | **Sum of Squares** | **DF** | **Mean Square** | ***F*-value** | ***p*-value** |  |
| --- | --- | --- | --- | --- | --- | --- |
| **Model** | 1.719E+05 | 4 | 42982.77 | 77.03 | < .0001 | **Significant** |
| **A-Peptone** | 10665.18 | 1 | 10665.18 | 19.11 | 0.0033 |  |
| **H-Temperature** | 87806.47 | 1 | 87806.47 | 157.35 | < .0001 |  |
| **J-Agitation** | 57001.80 | 1 | 57001.80 | 102.15 | < .0001 |  |
| **K-Inoculum Size** | 16457.61 | 1 | 16457.61 | 29.49 | 0.0010 |  |
| **Residual** | 3906.25 | 7 | 558.04 |  |  |  |
| **Total** | 1.758E+05 | 11 |  |  |  |  |

**(R^2^ = 0.9778; Adj R² =0.9651; Pred R² = 0.9347)**

**Table S 3***.* Analysis of variance (ANOVA) for *Kocuria* sp. RAM1 **carotenoids** using RSM.

| **Source** | **SS** | **df** | **Mean Square** | ***F*-value** | ***p*-value** |  |
| --- | --- | --- | --- | --- | --- | --- |
| **Model** | 1.116E+06 | 14 | 79729.66 | 13.60 | < .0001 | **Significant** |
| **A-Peptone** | 57174.08 | 1 | 57174.08 | 9.75 | 0.0070 |  |
| **B-Temperature** | 7.499E+05 | 1 | 7.499E+05 | 127.94 | < .0001 |  |
| **C-Agitation** | 1.383E+05 | 1 | 1.383E+05 | 23.60 | 0.0002 |  |
| **D-Inoculum Size** | 13351.94 | 1 | 13351.94 | 2.28 | 0.1520 |  |
| **AB** | 9942.08 | 1 | 9942.08 | 1.70 | 0.2124 |  |
| **AC** | 11491.84 | 1 | 11491.84 | 1.96 | 0.1818 |  |
| **AD** | 15770.34 | 1 | 15770.34 | 2.69 | 0.1217 |  |
| **BC** | 15013.60 | 1 | 15013.60 | 2.56 | 0.1303 |  |
| **BD** | 226.50 | 1 | 226.50 | 0.0386 | 0.8468 |  |
| **CD** | 1.64 | 1 | 1.64 | 0.0003 | 0.9869 |  |
| **A²** | 50927.95 | 1 | 50927.95 | 8.69 | 0.0100 |  |
| **B²** | 7072.27 | 1 | 7072.27 | 1.21 | 0.2893 |  |
| **C²** | 21511.68 | 1 | 21511.68 | 3.67 | 0.0747 |  |
| **D²** | 10892.03 | 1 | 10892.03 | 1.86 | 0.1929 |  |
| **Residual** | 87917.06 | 15 | 5861.14 |  |  |  |
| **Lack of Fit** | 65458.10 | 10 | 6545.81 | 1.46 | 0.3552 | not significant |
| **Pure Error** | 22458.96 | 5 | 4491.79 |  |  |  |
| **Cor Total** | 1.204E+06 | 29 |  |  |  |  |

**(R² = 0.9270; Adj R² = 0.8588; Pred R² = 0.6600; Adeq Precision = 14.6809)**
